# Place of Work and Level of Satisfaction with the Lives of Polish Nurses

**DOI:** 10.3390/nursrep10020013

**Published:** 2020-11-23

**Authors:** Anna Bartosiewicz, Małgorzata Nagórska

**Affiliations:** 1 Institute of Health Sciences, Medical College of Rzeszow University, 35-959 Rzeszow, Poland; abartosiewicz@ur.edu.pl; 2 Institute of Medical Sciences, Medical College of Rzeszow University, 35-959 Rzeszow, Poland

**Keywords:** work environment, life satisfaction, nurse

## Abstract

Practicing as a nurse may be a factor influencing the overall level of satisfaction with life. The aim of the study was to assess the level of satisfaction with nurses’ lives in relation to the place of employment. The research was conducted among nurses working in hospitals, primary health care, and outpatient specialist care. The study was carried out with the use of the satisfaction with life scale (SWLS) scale. Statistical analysis included a quantitative and qualitative approach to life satisfaction of the nurses surveyed. The impact of independent variables, measured on nominal (qualitative) scales on the results of the SWLS scale in quantitative terms, was assessed using one-way analysis of variance (ANOVA). Pairwise comparisons were assessed with the assumption of equality of variance with the Tukey honestly significant difference test. The level of satisfaction with life of the surveyed nurses was average. The place where nurses worked significantly influenced the level of life satisfaction. Nurses working in a hospital had a high level of satisfaction with life more so than nurses working in primary care or outpatient specialist care. The workplace is a factor that significantly differentiates the level of life satisfaction of the surveyed nurses.

## 1. Introduction

Most people spend an average one third of adult life at work [1], therefore professional activity, occupation, and working conditions may influence the general level of satisfaction with life. This also applies to nurses—the most numerous professional group amongst medical personnel [2]. That this profession is characterized by high demands, multi-tasking, and a high degree of difficulty [3] could be a reason of great satisfaction, but it is also burdened with great stress, and often leads to burnout [4,5,6]. The specificity of the profession means that a nurse can find a job both in hospitals and in open medical facilities. The article shows a relationship between the nurses’ workplace and their level of life satisfaction.

Satisfaction with life (SWL) is understood as a sense of satisfaction resulting from the expected results and relates to the cognitive process of every human being [7]. The level of life satisfaction, apart from the emotion of positive feelings and the lack of negative feelings, contributes to subjective well-being [8]. According to Juczyński, “the assessment of life satisfaction is the result of comparing your own situation with the standards you set. If the result of the comparison is satisfactory, then an appropriate level of satisfaction is received” [9]. Satisfaction is a long-term feeling and depends on many factors [10], such as health, work, family, social network, or financial situation [11]. Moreover, satisfaction with life is determined by other factors operating in different time perspectives, such as personality traits, previous life experiences, and mood at a given moment [12]. The topic can be justified by the many discussions resulting from the Word Health Organization (WHO) announcement of 2020 as the year of nurses. Many researchers’ results indicate that the factors determining the sense of life satisfaction among nurses are most often age, marital status, length of service, specificity of work, remuneration, emotional state, social support [13,14,15,16,17,18,19].

The aim of the study is to assess the level of satisfaction with nurses’ lives depending on the place of employment.

## 2. Material and Methods

The cross-sectional descriptive study was conducted in 2019 among nurses working in hospitals (H), primary health care (PHC), and outpatient specialist care (OSC) in the Podkarpackie Voivodeship south-east Poland. Invitations to participate in the study were sent to 37 medical entities, randomly selected via a randomized algorithm program. Sample size was determined with the help of the EPI INFO (StatCalc) software. A multistage random cluster sampling method was used. The message contained data on the planned research, 13 of them gave a positive feedback. The following criteria for the selection of respondents were adopted: Inclusion criteria—professionally active nurses working in H, PHC, and OSC, with at least three years of work experience, willing to participate in the survey; exclusion criteria—nurses with lower work experience, working in medical facilities other than those indicated above, nurses who did not consent to participate in the study, and incomplete questionnaires. Nurses working at the institutions that agreed to participate in the study were fully informed in writing and verbally about the nature of the study. They were assured of the voluntary participation in the survey and the anonymity of the answers provided. The survey questionnaires along with the consent form were delivered to the facilities. Participation in the study was voluntary and anonymous. To ensure the confidentiality of the data, the questionnaires were numbered and returned in sealed envelopes attached to the questionnaires, the correct completion of which was equivalent to the nurses expressing their participation in the study. Finally, 2740 questionnaires were distributed, 1286 (47%) were collected back, and 75 questionnaires were rejected due to incomplete responses. Data from 1211 questionnaires were analyzed using statistical methods. The study group included nurses working in hospitals (*n* = 405), primary health care (*n* = 414), and outpatient specialist care (*n* = 392).

The method used was a diagnostic survey using the survey technique. The participants of the study filled out socio-demographic data (age, seniority, education, additional qualifications, and position held) and the life satisfaction scale of the SWLS.

Life satisfaction scale: SWLS—the satisfaction with life scale, (E. Diener, R. A. Emmons, R.J. Larson, and S. Griffin) [10]. The scale contains five items rated on a seven-point scale. The respondent assesses to what extent each of the statements applies to their life so far. The object of measurement was the assessment of satisfaction with life, which results from comparing one’s own satisfaction with the standards set by oneself. The points were totaled and the obtained result, in the range from 5 to 35, determined the degree of satisfaction with life. When interpreting the results, one should follow the properties characterized by the sten scale. Scores within 1–4 sten were assumed to be low, and within 7–10 sten assumed to be high, which corresponds to the area of 33% of the lowest scores and the same number of the highest scores on the scale. Results within 5–6 sten are considered as average. Cronbach’s alpha reliability index is satisfactory and amounts to 0.81. The scale was adopted to Polish conditions [9].

### The Estimation Method and the Following Statistical Methods Were Used

The statistical analysis included a quantitative and qualitative approach to life satisfaction of the surveyed women. In quantitative terms, the dependent variable was included as a total value (5–35-point scale) and the value transcoded on the sten scale (1–10 points). The impact of independent variables, measured on nominal (qualitative) scales on the results of the SWLS scale in quantitative terms, was assessed using one-way analysis of variance (ANOVA). Pairwise comparisons were assessed with the assumption of equality of variance with the Tukey HSD (honestly significant difference) test. The reliability of the SWLS scale was assessed using the Cronbach’s alpha coefficient. The normality of the distribution of the dependent variable (SWLS) was assessed by the Kolmogorow–Smirnow and Shapiro–Wilk test. Obtaining the lack of normality of the distribution of variables prompted the researchers to qualify the results of life satisfaction, where the values obtained on the sten scale were grouped according to general rules (1–4 sten = low results, 5–6 sten = average results, 7–10 sten = high results). In this approach, the dependencies between the SWLS scale and independent variables were assessed with the Pearson χ^2^ test of independence. The significance level of the study was *p* < 0.05, while the calculations were made with the SPSS 20 package.

The study was approved by the institutional Bioethics Committee at the University of Rzeszów from 09/05/2019 (Resolution No. 30/05/2019) and by all appropriate administrative bodies.

## 3. Results

### 3.1. Characteristics of the Study Group

The study group consisted of 1211 nurses. The mean age of women was 44.56 ± 11.52 years, and the ages ranged between 22–69 years. Half of the respondents were under 47 years of age. The respondents more often lived in the city (56.58%). Almost half of the people (48.06%) had work experience over 25 years. Similarly, the level of education was secondary for almost half of the respondents (48.14%), and every third nurse had BA education (32.95%). Only every fifth respondent (22.21%) had a specialization. The groups were comparable in terms of the size of the workplace (χ^2^ = 0.606; df = 2; *p* = 0.7386). Almost all of the surveyed nurses (95.62%) did not hold a managerial position. Similarly, the majority of people (86.95%) had a family, three quarters of respondents assessed their financial situation as income equal to expenses. The respondents most often assessed their health condition as good (41.12%) or average (31.71%) (Table 1).

### 3.2. Findings

The reliability of the SWLS scale was high (Cronbach’s alpha 0.828). All the individual five questions (items) of the scale correlated positively with each other (in the range r = 0.416–0.605), as well as positively with the overall score of the scale (in the range r = 0.577–0.660).

The levels of satisfaction with the life of the respondents were most visible in terms of satisfaction with life (4.81 points) and satisfaction with achieving the most important things in life (4.45 points). The respondents thought to the least extent that their lives were in many respects close to ideal (3.64 points) or that they had excellent living conditions (3.69 points).

The table below shows the percentages, numbers, means (scale 1–7 points), standard deviation, and variance (variability of results)—this allows us to conclude that the nurses differed most in the question about the possibility of living their life again the same (2.66), and the smallest discrepancy was noticed in the question about life satisfaction (1.55), (Table 2).

**Figure 1:**
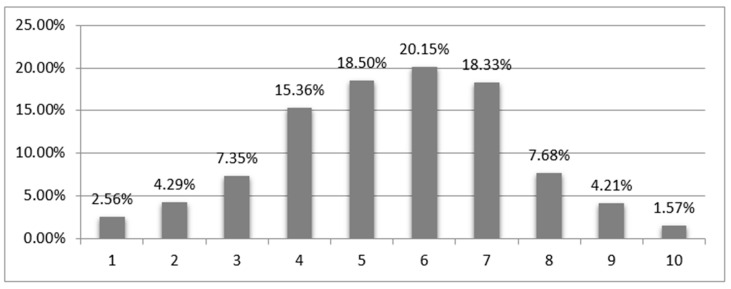
Level of life satisfaction (SWLS) according to the sten scale (1–10 points).

The mean SWLS level in the raw scores was 20.46 ± 5.48 points, and fluctuated across the scale (5–35 points), so there were people who were completely dissatisfied with the quality of their lives, as well as people who were fully satisfied. Translating the raw results to the sten scale showed that the respondents most often obtained results between 4–7 points (72.34% of respondents in total had results in this range, and the remaining nurses scored below 4 points or above 7 points), (Figure 1).

Low results on the SWLS were obtained by 29.56% of the nurses (*n* = 358). 38.65% of people (*n* = 468) had high results and this was the largest group of respondents. A high level of satisfaction with life concerned 31.79% of the respondents (*n* = 385). The differences in the frequency of the occurrence of the three groups of the quality of life satisfaction level were statistically significant (χ^2^ = 16.282; df = 2, *p* = 0.0003).

### 3.3. Satisfaction with Life Scale (SWLS) by Variables for All Respondents (n = 1211)

The low level of satisfaction with life was more common in the age groups 41–50 (36.9%) and over 50 (32.3%). The results at the average level were comparable in all age groups. The high level of satisfaction with life is presented more often by nurses under 30 (49.5%) and in the age range of 31–40 (38.2%). The differences were statistically significant (*p* < 0.0001).

The nurses’ place of residence did not significantly (*p* = 0.0605) affect the level of life satisfaction. More frequent occurrence of a high level of satisfaction with life was observed among rural residents (35.1%) than among urban residents (29.0%).

Work experience in the profession significantly (*p* < 0.0001) differentiated the level of satisfaction with the life of nurses. It has been shown that nurses with 11–25 years (35.6%) or over 25 (32.8%) years of service were more likely to have a low level of life satisfaction. The average satisfaction with life was shared with equal frequency by all groups of seniority. A high level of satisfaction with life is the domain of more frequent respondents with work experience of up to 10 years (47.1%) than respondents with work experience over 10 years.

The level of education significantly (*p* < 0.0001) influenced the satisfaction with life of the respondents. Low level of satisfaction was more common among nurses with secondary nursing education (35.2%), less often those with first-cycle studies (bachelor’s level) (27.3%) or second-cycle studies (master’s level) (19.2%). The differentiation of the average level of satisfaction with life due to the level of education of the respondents was not significant. The average level of life satisfaction declared mainly nurses with secondary nursing education (40.8%) or nurses on a master’s level (37.6%), next those with bachelor’s level (36.1%). A high level of satisfaction with life was observed in respondents with higher education-first-cycle studies (bachelor’s level) (36.6%) or second-cycle studies (master’s level) (43.2%), much less often (24.0%) those with secondary education.

A high level of satisfaction with life was more common (37.9%) among nurses with specialization than those without specialization (30.0%). On the other hand, low quality of life was reported more often by nurses without specialization (31.0%) than those with specialization (24.5%). The differences were statistically significant (*p* = 0.0281).

The place where nurses worked (*p* < 0.0001) significantly influenced the level of life satisfaction. Low level of satisfaction was more often reported by PHC (34.3%) and OSC (31.9%), compared to nurses working in a hospital (22.5%). The average level of satisfaction with life did not differ depending on the place of residence. On the other hand, high level of satisfaction with life was reported more often (42.7%) by nurses working in a hospital than nurses working in PHC (24.6%) or OSC (28.1%).

Nurses with a low level of satisfaction with life were more often people who did not occupy a managerial position (30.2%), while high satisfaction with life was more often (56.6%) among nurses holding a managerial position. The differences were statistically significant (*p* = 0.0003).

Having a family did not significantly (*p* = 0.1001) affect the level of women’s satisfaction with life.

The financial situation significantly differentiated the satisfaction with life of nurses (*p* = 0.0077). Low level of satisfaction with life more often concerned people whose income was lower than expenditure (30.1%) or equal to expenditure (30.7%). On the other hand, a high level of satisfaction with life was observed more often by respondents who had an income higher than expenditure (47.4%).

Nurses with average (37.0%) or unsatisfactory (36.4%) health condition had a low level of satisfaction with life. On the other hand, the respondents who assessed their health condition as very good (44.1%) or good (34.5%) had a high level of satisfaction with life. The differences were statistically significant (*p* < 0.0001).

### 3.4. Differences between Nurses Working in Primary Health Care, Outpatient Specialist Care, or in Hospital and the Level of Life Satisfaction in Individual Categories of Independent Variables

It was shown that differences between the level of satisfaction with life and the place of work were visible in two age groups—under 30 (*p* = 0.0030) and over 50 (*p* = 0.0362). In the age group <30 years, a high level of life satisfaction was found more often in H nurses (55.7%), less often in OSC (44.1%), and the least (29.7%) in primary care nurses. In the case of women over 50 years of age, a high level of life satisfaction was found more often than in comparison with other nurses working in the hospital (37.8%) than in PHC or OSC employees (21.7% each). There were no statistically significant differences between the place of work and the level of job satisfaction in the age groups 31–40 (*p* = 0.3341) and 41–50 (*p* = 0.3963) (Table 3).

Differences between the place of work and the level of life satisfaction were noticeable among nurses with first-cycle studies (*p* = 0.0003) and second-cycle studies (*p* = 0.0176). In the group of respondents with a bachelor’s degree in nursing, a high level of life satisfaction was found more often among nurses working in the hospital (47.6%), less often among OSC employees (30.6%), and to the least extent among primary care nurses (23.2%). A similar relationship was observed among nurses with a master’s degree, where a high level of life satisfaction was reported by, respectively, 47.6% hospital, 43.0% OSC, and 30.4% primary care (Table 4).

The place of work significantly influenced the level of life satisfaction of both nurses with a family (*p* < 0.0001) and nurses without a family (*p* = 0.0219). Among women with a family, a high level of life satisfaction was found more often among nurses working in a hospital (40.7%), and less often among employees of primary care (23.9%) or OSC (29.0%). In the group of women who did not have a family, the relation was similar—female employees of the hospital (50.6%) had a high level of life satisfaction more often, and less often those of primary care (32.4%) or OSC (20.0%) (Table 5).

## 4. Discussion

The level of satisfaction with life is influenced by many external and internal factors. The article takes the level of life satisfaction among the surveyed nurses depending on the workplace and socio-demographic factors. The paper analyzes the level of life satisfaction of professionally active nurses depending on the place of employment. The SWLS score was 20.46 and is an assessment of the average level of life satisfaction in the surveyed group of nurses. Similar results were obtained in most studies by other Polish authors: Kliszcz (19.57), Wysokiński (19.60), Kupcewicz (21.00), Swatowska (20.66), Pietraszek (21.10), and Lewko (21.50) [13,15,20,21,22,23]. Only in the work by Jakubowska was the result variant; 23.17, i.e., a high level of satisfaction with life [14]. For comparison, in studies among nurses in Iran, the SWLS score was 16.36, and in Korea, 22.00 [17,18].

In our study we took into account place of work as a determinant of the SWL level. As confirmed by the research, the workplace was a significant influence on the level of satisfaction. Nurses working in hospitals with a bachelor’s degree and not in a managerial position have a higher level of life satisfaction than nurses working for PHC and OSC. The level of satisfaction was highest in hospital nurses, slightly lower in OSC, and the lowest among PHC workers. On the other hand, in the study by Wysokiński, the highest SWL level was reported by nurses working in clinics [15]. Similarly, for Pietraszek, where the higher SWL level was confirmed by nurses employed in PHC [22]. As it turns out, among the nurses employed in hospitals, the department where the respondents worked had an impact on the SWL level [14]. The SWL level is also influenced by the nature of work, as it turns out that where nurses have direct contact with the patient, the SWL level is lower [22]; in contrast, the results of our research indicate that nurses struggling in direct contact with the patient show a higher level of satisfaction with life.

Many authors analyzed the influence of socio-demographic and occupational factors on SWL. Most of them indicate that SWL changes positively with age. In the work of Jakubowska, the highest SWL level was recorded in the 35–44 age group, while in Wysokiński over 50 years old, which was partially confirmed in her own research, where the highest SWL level was assessed by nurses in the age groups over 50 and under 30 [15,24]. The obtained results showed a higher SWL level among rural residents, as in Jakubowski’s case, while in Wysokiński’s study it was city residents who indicated a higher SWL level [14,15]. However, according to Dziąbek, the place of residence did not affect significantly the SWL level [25]. The high level of SWL was declared by people employed longer (over 10 years), which was confirmed by the results of other researchers [15,16], different than Dziąbek, where “the longer nurses work, the level of SWL is lower” [25]. Higher SWL level was more often declared by respondents with higher education, and the lowest with secondary education, as in the works of Jakubowska, Swatowska, and Kupcewicz [14,20,21,26]. Only Wysokiński had a higher SWL level of nurses with secondary education, i.e., graduates of medical secondary schools and post-secondary schools [15]. Additionally, our own research confirmed the positive impact of having a specialization on SWL. Pietraszek et al. showed that holding a managerial position gives a higher sense of SWL [22], which was also confirmed in our research. Similarly, in Korean studies higher life achievements related to professional work positively influenced SWL [18]. The life situation also affects the level of satisfaction with life. Most authors found that married nurses had a higher level of satisfaction with life than single people [14,15,16,19,20,21]. This relationship was not confirmed in our study. Additionally, in our work, we took into account the experience of motherhood as one of the factors determining the sense of SWL and its higher level was declared by childless people.

The results of studies by Kupcewicz, Ghazwin, and own research confirm that the overall level of life satisfaction is influenced by the financial situation [17,20,23]. A few of the authors have considered health as a determinant of overall life satisfaction. Such confirmation, apart from our analysis, was obtained from Kliszcz and Pietraszek [13,22]. Additionally, Kliszcz indicates the emotional situation, i.e., the level of anxiety and depression, as a dependent condition for SWL. In turn, the level of empathy as a variable determining the level of satisfaction with life was indicated by Caro et al. [27].

As confirmed by the research, the level of satisfaction with life is the resultant of many socio-demographic factors such as age, place of residence, having one’s own family, financial and health situation, as well as professional factors such as level of education, workplace, and position held. The limitation of the study undertaken was that it was carried out only in a group of female nurses and in one region of Poland. The analysis carried out focused solely on the socio-demographic and professional factors. In the future, it would be worth extending the study and learning about the dependence of SWL on internal factors such as personality type, life experience, or worldview.

## 5. Conclusions

The surveyed nurses present an average level of satisfaction with life. The workplace of nurses significantly differentiates the level of life satisfaction—the level was higher among nurses working in a hospital than among nurses working in primary health care and outpatient specialist care. Socio-demographic factors such as age, length of service, place of residence, having one’s own family, financial and health situation, as well as professional factors such as level of education, workplace, and the position held significantly affect the level of life satisfaction of the surveyed nurses.

## Figures and Tables

**Table 1:** Characteristics of the studied group.

Independent Variables	Categories	*n*	%
Age	Up to 30 years	220	18.17
	From 31–40 years old	165	13.63
	From 41–50 years old	393	32.45
	From 51–60 years old	388	32.04
	Over 60 years	45	3.72
Place of residence	Town	661	54.58
	Village	550	45.42
Work experience as a nurse	From 1–5 years	201	16.60
	From 6–10 years	111	9.17
	From 11–15 years	104	8.59
	From 16–24 years	213	17.59
	Over 25 years	582	48.06
Education	Secondary nursing education	583	48.14
	Bachelor’s degree	399	32.95
	Master’s degree	229	18.91
Additional qualifications	No	942	77.79
	Yes	269	22.21
Place of work	Primary health care (PHC)	414	34.19
	Hospital (H)	405	33.44
	Outpatient specialist care (OSC)	392	32.37
Held position	Not managerial	1158	95.62
	Managerial	53	4.38
Having a family/motherhood experience	No	158	13.05
	Yes	1053	86.95
Self-assessment of the material situation	Revenues lower than expenses	209	17.26
	Revenues equal to expenses	907	74.90
	Revenues higher than expenses	95	7.84
Self-assessment of health	Very good	263	21.72
	Good	498	41.12
	Average	384	31.71
	Disappointing	66	5.45

**Table 2:** Level of life satisfaction in relation to individual items of the scale.

SWLS/Items		Item No 1	Item No 2	Item No 3	Item No 4	Item No 5
I totally disagree	*n*	99	68	21	31	106
	%	8.2	5.6	1.7	2.6	8.8
I do not agree	*n*	169	188	45	106	190
	%	14.0	15.5	3.7	8.8	15.7
Rather disagree	*n*	250	293	99	155	202
	%	20.6	24.2	8.2	12.8	16.7
Neither agree nor agree	*n*	363	299	237	250	228
	%	30.0	24.7	19.6	20.6	18.8
I rather agree	*n*	222	258	446	386	286
	%	18.3	21.3	36.8	31.9	23.6
I agree	*n*	91	79	302	225	150
	%	7.5	6.5	24.9	18.6	12.4
Totally agree	*n*	17	26	61	58	49
	%	1.4	2.1	5.0	4.8	4.0
Average		3.64	3.69	4.81	4.45	3.86
SD		1.41	1.39	1.25	1.41	1.63
Variance		1.99	1.94	1.55	2.00	2.66

Item No 1: In many ways, my life is close to ideal; Item No 2: The conditions of my life are perfect; Item No 3: I am happy with my life; Item No 4: I’ve achieved the most important things I wanted in my life; Item No 5: If I could live my life again, I would hardly change anything.

**Table 3:** Place of work and level of life satisfaction depending on age, place of residence, and seniority.

Independent Variables				Place of Work			*p*
				PHC	H	OSC	
Age: Less than 30 years	Level of satisfaction with life	low	*n*	11	14	9	0.0030
			%	29.7	9.4	26.5	
		average	*n*	15	52	10	
			%	40.5	34.9	29.4	
		high	*n*	11	83	15	
			%	29.7	55.7	44.1	
	Total			37	149	34	
			%	100.0	100.0	100.0	
Age: Over 50 years	Level of satisfaction with life	low	*n*	61	21	58	0.0362
			%	33.9	24.1	34.9	
		average	*n*	80	33	72	
			%	44.4	37.9	43.4	
		high	*n*	39	33	36	
			%	21.7	37.9	21.7	
	Total			180	87	166	
			%	100.0	100.0	100.0	
Place of residence: Town	Level of satisfaction with life	low	*n*	78	35	85	0.0003
			%	32.8	20.5	33.7	
		average	*n*	100	64	107	
			%	42.0	37.4	42.5	
		high	*n*	60	72	60	
			%	25.2	42.1	23.8	
	Total			238	171	252	
			%	100.0	100.0	100.0	
Place of residence: Village	Level of satisfaction with life	low	*n*	64	56	40	0.0016
			%	36.4	23.9	28.6	
		average	*n*	70	77	50	
			%	39.8	32.9	35.7	
		high	*n*	42	101	50	
			%	23.9	43.2	35.7	
	Total			176	234	140	
			%	100.0	100.0	100.0	
Seniority: 1–5 years	Level of satisfaction with life	low	*n*	9	16	8	0.0194
			%	32.1	11.2	26.7	
		average	*n*	10	48	10	
			%	35.7	33.6	33.3	
		high	*n*	9	79	12	
			%	32.1	55.2	40.0	
	Total			28	143	30	
			%	100.0	100.0	100.0	
Seniority: 16–25 years	Level of satisfaction with life	low	*n*	37	21	22	0.0463
			%	41.6	43.8	28.9	
		average	*n*	36	11	29	
			%	40.4	22.9	38.2	
		high	*n*	16	16	25	
			%	18.0	33.3	32.9	
	total		*n*	89	48	76	
			%	100.0	100.0	100.0	

PHC—Primary Health Care; H—Hospital, OSC—Outpatient Specialist Care.

**Table 4:** Place of work and the level of life satisfaction depending on education, specialization, and position held.

Independent Variables				Place of Work			*p*
				PHC	H	OSC	
Education: Bachelor’s degree	Level of satisfaction with life	low	*n*	40	39	30	0.0003
			%	35.7	20.6	30.6	
		average	*n*	46	60	38	
			%	41.1	31.7	38.8	
		high	*n*	26	90	30	
			%	23.2	47.6	30.6	
	Total		*n*	112	189	98	
			%	100.0	100.0	100.0	
Education: Master’ degree	Level of satisfaction with life	low	*n*	19	7	18	0.0176
			%	27.5	9.5	20.9	
		average	*n*	29	26	31	
			%	42.0	35.1	36.0	
		high	*n*	21	41	37	
			%	30.4	55.4	43.0	
	Total		*n*	69	74	86	
			%	100.0	100.0	100.0	
No additional qualification	Level of satisfaction with life	low	*n*	119	68	105	<0.0001
			%	36.6	22.4	33.4	
		average	*n*	128	103	136	
			%	39.4	34.0	43.3	
		high	*n*	78	132	73	
			%	24.0	43.6	23.2	
	Total		*n*	325	303	314	
			%	100.0	100.0	100.0	
Additional qualification	Level of satisfaction with life	low	*n*	23	23	20	0.0470
			%	25.8	22.5	25.6	
		average	*n*	42	38	21	
			%	47.2	37.3	26.9	
		high	*n*	24	41	37	
			%	27.0	40.2	47.4	
	Total		*n*	89	102	78	
			%	100.0	100.0	100.0	
Position held: Not managerial	Level of satisfaction with life	low	*n*	139	88	123	<0.0001
			%	34.8	23.1	32.6	
		average	*n*	163	135	155	
			%	40.8	35.4	41.1	
		high	*n*	98	158	99	
			%	24.5	41.5	26.3	
	Total		*n*	400	381	377	
			%	100.0	100.0	100.0	

PHC—Primary Health Care; H—Hospital, OSC—Outpatient Specialist Care.

**Table 5:** Place of work and level of life satisfaction depending on family ownership, financial situation, and self-assessment of health.

Independent Variables				Place of Work			*p*
				PHC	H	OSC	
I don’t have a family	Level of satisfaction with life	low	*n*	10	15	13	0.0219
			%	27.0	18.5	32.5	
		average	*n*	15	25	19	
			%	40.5	30.9	47.5	
		high	*n*	12	41	8	
			%	32.4	50.6	20.0	
	Total			37	81	40	
				100.0	100.0	100.0	
I have a family	Level of satisfaction with life	low	*n*	132	76	112	<0.0001
			%	35.0	23.5	31.8	
		average	*n*	155	116	138	
			%	41.1	35.8	39.2	
		high	*n*	90	132	102	
			%	23.9	40.7	29.0	
	Total			377	324	352	
				100.0	100.0	100.0	
Financial situation: Revenues equal to expenses	Level of satisfaction with life	low	*n*	125	45	108	<0.0001
			%	35.2	19.8	33.2	
		average	*n*	152	77	129	
			%	42.8	33.9	39.7	
		high	*n*	78	105	88	
			%	22.0	46.3	27.1	
	Total			355	227	325	
				100.0	100.0	100.0	
Health self-assessment: Good	Level of satisfaction with life	low	*n*	65	17	53	0.0026
			%	31.0	17.0	28.2	
		average	*n*	84	32	75	
			%	40.0	32.0	39.9	
		high	*n*	61	51	60	
			%	29.0	51.0	31.9	
	Total			210	100	188	
				100.0	100.0	100.0	
Health self-assessment: Average	Level of satisfaction with life	low	*n*	64	24	54	0.0008
			%	40.5	28.2	38.3	
		average	*n*	68	30	67	
			%	43.0	35.3	47.5	
		high	*n*	26	31	20	
			%	16.5	36.5	14.2	
	Total			158	85	141	
			%	100.0	100.0	100.0	

PHC—Primary Health Care; H—Hospital, OSC—Outpatient Specialist Care.
